# Chest wall reconstruction with titanium plates after desmoid tumor resection

**DOI:** 10.1590/S1806-37132014000200017

**Published:** 2014

**Authors:** Fernando Luiz Westphal, Luís Carlos de Lima, José Corrêa Lima Netto, Stephany da Cunha Seelig, Katienne Frota de Lima

**Affiliations:** Federal University of Amazonas School of Medicine Getúlio Vargas University Hospital, Manaus, Brazil; Department of Thoracic Surgery, Federal University of Amazonas School of Medicine Getúlio Vargas University Hospital, Manaus, Brazil; Federal University of Amazonas School of Medicine, Manaus, Brazil; Federal University of Amazonas School of Medicine, Manaus, Brazil

## To the Editor:

Chest wall reconstruction becomes necessary when there are wall defects larger than 5 cm in diameter that compromise respiratory dynamics. Its purpose is to restore wall integrity, as well as to maintain waterproofing of the pleura, an aesthetic chest contour, and respiratory dynamics. In addition, the purpose is to protect vital intrathoracic organs, thus preventing lung herniation and paradoxical breathing and preserving lung compliance.^(^ ^1^ ^,^ ^2^ ^)^

The indication for bony reconstruction of the chest wall is related to the size and location of the defect. Defects in the anterior, lateral, and sternal wall require reconstruction, whereas defects in the posterior wall can be covered by the posterior muscles or by the scapula and do not require the use of prostheses.^(^ ^1^ ^)^

There is as yet no consensus on the ideal material for use in rib reconstruction. The literature suggests the use of prostheses consisting of titanium plates (STRATOS^TM^, Strasbourg Thoracic Osteosynthesis System; Diagnostic Medical Systems, Pérols, France) for that purpose, and, therefore, we report the case of a 25-year-old female patient who presented with a nearly one-year history of chest pain and dyspnea, as well as with a volume increase in the left costal margin.

Physical examination revealed a tumor in the lower third of the anterior chest wall, affecting the left thoracoabdominal junction. An axial CT scan of the chest showed a soft-tissue tumor that affected the region of the left anterior costal margin, extended to the abdominal region, and compressed the left hepatic lobe, the anterior pericardium, and the lung parenchyma in the left lower lobe. However, there were no signs of structural invasion. The tumor measured 12.0 × 11.0 × 7.5 cm.

**Figure 1:**
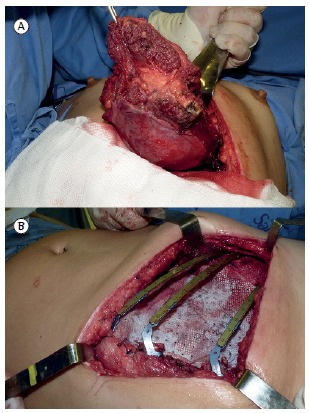
Images of the desmoid tumor resection and chest wall reconstruction. In A, exposure of the tumor of the left costal margin during the surgical procedure, including ribs, muscles, the distal portion of the sternum, and the diaphragm. In B, chest wall reconstruction with a two-layered mesh and titanium bars.

The patient underwent chest wall resection, which included soft tissues and the anterior portion of the sixth, seventh, and eighth ribs, as well as the costal margin (Figure 1A). Histopathological examination of the tumor showed that it was a desmoid tumor-a rare, benign, unencapsulated neoplasm with strong infiltrative capability locally and a high rate of recurrence after surgical resection.^(^ ^3^ ^)^ Chest wall reconstruction was performed with PHYSIOMESH^TM^ (ETHICON^(r)^; Johnson & Johnson, Somerville, NJ, USA) and three titanium plates (STRATOS^TM^; Figure 1B).

The ideal material for reconstruction should have the following characteristics: being adaptable; being durable; being transparent to X-ray; causing minimal inflammatory reaction; and being resistant to infection. Typically, the materials used are nylon, silicone, acrylic, Silastic^(r)^ (Dow Corning Corp., Midland, MI, USA), Prolene^(r)^ mesh, Vicryl^(r)^ mesh (polygalactin; ETHICON^(r))^, Gore-Tex^(r)^ (polytetrafluoroethylene; Gore Company, Flagstaff, AZ, USA), and Marlex mesh (polypropylene).^(^ ^1^ ^,^ ^2^ ^)^

Currently, Marlex mesh is the most widely used material, because it is easy to handle, permeable, highly resistant, durable, and inexpensive. In addition, it is hardly susceptible to infection. However, in contact with the lung, it causes adhesions and an intense fibrotic reaction hindering possible thoracic reoperations, as well as not providing proper support for the chest wall.^(^ ^2^ ^)^

The mesh used in our patient (PHYSIOMESH^TM)^ is composed of two layers: a Monocryl^(r)^ film (polyglecaprone 25), which is partially absorbable and reduces adhesion to the visceral organs (in the present case, the lung, the diaphragm, and the pericardium), thus facilitating performing another surgical intervention if necessary; and a Prolene^(r)^ film (polypropylene), which is consistent with the required resistance for the chest wall, thus providing comfortable healing. This mesh is placed between the lung and the titanium prostheses, thus preventing lung herniation and protecting the lung from contact with the plates.

STRATOS^TM^ consists of titanium bars and clips that form a vertical expandable prosthetic system. It has recently been used for fixation of rib fractures and for chest reconstruction after tumor resection.^(^ ^4^ ^)^

The titanium plates, once integrated into the chest wall, will form an oxide layer that is highly resistant to corrosion. They have the highest strength-to-weight ratio among all metals, i.e., titanium plates have low weight but have stiffness similar to that of the ribs. The titanium plates have the facility of integrating with the bones, which prevents detachment from the ribs over time, and are highly resistant to infections. They do not interfere with imaging or preclude magnetic resonance imaging.^(^ ^5^ ^)^

Previously published reports of patients who were operated on and received STRATOS^TM^ have shown that its material does not affect the chances of local tumor recurrence. One group of authors used STRATOS^TM^ in a male patient with an Ewing's sarcoma of proportions similar to those of the tumor in our patient, and, after a 21-month follow-up period, the patient had no tumor recurrence.^(^ ^6^ ^)^

**Figure 2:**
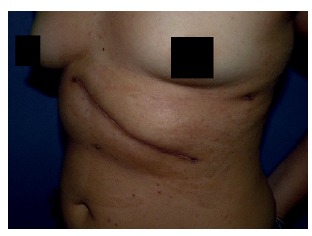
Patient appearance in the sixth postoperative month. Note that the contour of the costal margin was maintained, being stable and symmetrical.

Although there are no studies that define STRATOS^TM^ as the ideal system for use in chest wall reconstruction, it is technically simple and well tolerated. In addition, other case reports and articles comparing this system with older techniques have reported better restoration of the contour of the ribs (Figure 2) and preservation of respiratory mechanics, as well as greater comfort. Maintaining chest wall symmetry prevents localized chest deformity, as well as the scoliosis seen over time in patients with a partially collapsed chest.^(^ ^4^ ^,^ ^6^ ^)^

We emphasize the importance of the present report, given that, to our knowledge, it is the first such report in the Brazilian literature. The existing option of a substitute for ribs increases the chances of major chest wall resections, which is an important factor in treating tumors with oncologic margins.
