# Supplementary material for: Chest wall reconstruction with titanium plates after desmoid tumor resection
Source: J Bras Pneumol. 2014 Mar-Apr;40(2):200–2. doi: 10.1590/S1806-37132014000200017 (PMC4083637; doi:10.1590/S1806-37132014000200017)
Supplement: Translation

# Translation: Reconstrução de parede torácica com placas de titânio após ressecção de tumor desmoide

**Authors:** Fernando Luiz Westphal, Luís Carlos de Lima, José Corrêa Lima Netto, Stephany da Cunha Seelig, Katienne Frota de Lima

**Affiliations:** Hospital Universitário Getúlio Vargas, Faculdade de Medicina, Universidade Federal do Amazonas, Manaus (AM) Brasil; Hospital Universitário Getúlio Vargas, Faculdade de Medicina, Universidade Federal do Amazonas, Manaus (AM) Brasil; Serviço de Cirurgia Torácica, Hospital Universitário Getúlio Vargas, Faculdade de Medicina, Universidade Federal do Amazonas, Manaus (AM) Brasil; Faculdade de Medicina, Universidade Federal do Amazonas, Manaus (AM) Brasil; Faculdade de Medicina, Universidade Federal do Amazonas, Manaus (AM) Brasil

## Ao Editor:

A reconstrução da parede torácica se torna necessária quando há defeitos de parede com diâmetro maior que 5 cm que comprometam a dinâmica respiratória. Seu objetivo é restaurar a integridade da parede, assim como manter a impermeabilização pleural, o padrão estético do contorno do tórax e a dinâmica respiratória. Além disso, objetiva-se proteger os órgãos vitais intratorácicos, evitando a herniação pulmonar e a respiração paradoxal e preservando a complacência pulmonar.^(^ ^1^ ^,^ ^2^ ^)^

A indicação para a reconstrução óssea da parede torácica tem relação com o tamanho e a localização do defeito. Em defeitos na parede anterior, lateral e esternal, torna-se necessária a reconstrução, enquanto defeitos na parede posterior podem ser recobertos pelos músculos posteriores ou escápula, sendo desnecessária a utilização de próteses.^(^ ^1^ ^)^

Ainda não há consenso quanto ao material ideal a ser utilizado em reconstrução de arcos costais. A literatura sugere a utilização da prótese de placas de titânio (STRATOS^TM^, Strasbourg Thoracic Osteosynthesis System; Diagnostic Medical Systems, Pérols, França) para essa finalidade e, por tal motivo, relatamos o caso de uma paciente do sexo feminino, 25 anos, com queixa de dor torácica e dispneia discreta há cerca de um ano, relatando um aumento de volume do rebordo costal esquerdo.

Ao exame físico, foi observada uma tumoração em terço inferior da parede torácica anterior, com comprometimento da transição toracoabdominal à esquerda. A TC axial do tórax demonstrou uma tumoração de partes moles comprometendo a região do rebordo costal anterior esquerdo, com extensão para região abdominal e compressão do lobo hepático esquerdo, pericárdio anterior e parênquima pulmonar do lobo inferior esquerdo; entretanto, sem sinais de invasão das estruturas. A tumoração media 12,0 × 11,0 × 7,5 cm.

A paciente foi submetida à ressecção da parede torácica, que incluiu tecidos moles e a porção anterior dos 6º, 7º e 8º arcos costais e o rebordo costal (Figura 1A). A análise histopatológica do tumor demonstrou ser um tumor desmoide - neoplasia benigna rara, não encapsulada, com alto poder infiltrativo local e alta taxa de recidiva após ressecção cirúrgica.^(^ ^3^ ^)^ A reconstrução da parede torácica foi realizada com tela PHYSIOMESH^TM^ (ETHICON^(r)^; Johnson & Johnson, Somerville, NJ, EUA) e três placas de titânio, o STRATOS^TM^ (Figura 1B).

**Figura 1:** Imagens da ressecção de tumor desmoide e reconstrução de parede torácica. Em A, tumoração do rebordo costal esquerdo exposta durante o ato operatório, incluindo arcos costais, músculos, porção distal do esterno e diafragma. Em B, reconstrução da parede com tela de duas camadas e barras de titânio.

O material ideal para a reconstrução deve apresentar as seguintes características: adaptabilidade, durabilidade, transparência ao raio X, mínima reação inflamatória e resistência à infecção. Os materiais geralmente utilizados são nylon, silicone, acrílico, Silastic^(r)^ (Dow Corning Corp., Midland, MI, EUA), tela de Prolene^(r)^, tela de Vicryl^(r)^ (poligalactina; ETHICON^(r))^, Gore-Tex^(r)^ (politetrafluoretileno; Gore Company, Flagstaff, AZ, EUA) e tela de Marlex (polipropileno).^(^ ^1^ ^,^ ^2^ ^)^

As telas de Marlex são os materiais mais utilizados atualmente, pois são fáceis de manipular, permeáveis e possuem alta resistência, durabilidade e baixo custo; além disso, são pouco suscetíveis à infecção. Porém, em contato com o pulmão, promovem aderências e intensa reação fibrótica que dificulta possíveis reoperações torácicas, além de não propiciarem uma adequada sustentação da parede torácica.^(^ ^2^ ^)^

A tela utilizada em nossa paciente (PHYSIOMESH^TM)^ é composta por duas camadas: uma película de Monocryl^(r)^ (poliglecaprone 25), que é parcialmente absorvível e reduz a aderência aos órgãos viscerais (no presente caso, o pulmão, o diafragma e o pericárdio), facilitando uma nova intervenção cirúrgica caso seja necessária; e uma película de Prolene^(r)^ (polipropileno), compatível com a resistência necessária para a parede torácica, proporcionando uma cicatrização confortável. Essa tela é colocada entre o pulmão e as próteses de titânio, evitando a herniação pulmonar e protegendo o pulmão do contato com as placas.

O STRATOS^TM^ é composto por grampos e barras de titânio, que formam um sistema protético vertical expansível. Tem sido utilizado recentemente para a fixação de fraturas de costela e na reconstrução torácica após a ressecção de tumores.^(^ ^4^ ^)^

As placas de titânio, quando integradas à parede torácica, formam uma camada do óxido altamente resistente à corrosão. Possuem a maior relação força-peso dentre os metais, ou seja, têm menor peso, mas mantêm uma rigidez semelhante à dos arcos costais. Apresentam a facilidade de integrar-se com os ossos, impedindo o desprendimento das costelas ao longo do tempo, e possuem maior resistência a infecções. Não causam interferência em exames de imagem e não impedem a realização de ressonância nuclear magnética.^(^ ^5^ ^)^

Os casos já publicados de pacientes que foram operados e receberam o STRATOS^TM^ demonstraram que seu material não interfere na chance de recorrência de neoplasias no local. Alguns autores utilizaram o STRATOS^TM^ em um paciente com sarcoma de Ewing de proporções semelhantes ao tumor em nossa paciente e, após 21 meses de acompanhamento, o paciente não apresentou recorrência da neoplasia.^(^ ^6^ ^)^

Não há estudos que definam o STRATOS^TM^ como o método ideal na reconstrução de parede torácica; porém, esse é tecnicamente simples e bem tolerado. Além disso, outros relatos de caso descritos e artigos que comparam esse sistema com as técnicas mais antigas relatam uma melhor restauração do contorno dos arcos costais (Figura 2), preservação da mecânica da respiração e maior conforto. A manutenção da simetria da parede torácica evita a deformidade torácica local, assim como a escoliose observada em longo prazo em pacientes com colapso de uma das porções do tórax.^(4,6)^

**Figura 2:** Aspecto da paciente no sexto mês de pós-operatório. Observa-se contorno do rebordo costal mantido, estável e simétrico.

Salientamos a importância do presente relato, visto que é o primeiro descrito na literatura nacional pelo que sabemos. A opção de um substituto para os arcos costais amplia as chances de ressecções maiores da parede torácica, fato importante para o tratamento de tumores com margens oncológicas.
